# *Astragalus mongholicus* Bunge and *Curcuma aromatica* Salisb. modulate gut microbiome and bile acid metabolism to inhibit colon cancer progression

**DOI:** 10.3389/fmicb.2024.1395634

**Published:** 2024-06-17

**Authors:** Xu Wang, Boyang Zhu, Yongzhi Hua, Ruolan Sun, Xiying Tan, Xiangwei Chang, Decai Tang, Junfei Gu

**Affiliations:** ^1^School of Traditional Chinese Medicine, Nanjing University of Chinese Medicine, Nanjing, China; ^2^College of Pharmacy, Anhui University of Chinese Medicine, Hefei, China; ^3^Affiliated Hospital of Integrated Traditional Chinese and Western Medicine, Nanjing University of Chinese Medicine, Nanjing, China; ^4^Affiliated Hospital of Nanjing University of Traditional Chinese Medicine, Nanjing, China

**Keywords:** colon cancer, gut microbiome, bile acid, *Astragalus mongholicus* Bunge and *Curcuma aromatica* Salisb., FabG, baiA

## Abstract

**Introduction:**

Alterations in the gut microbiome and bile acid metabolism are known to play a role in the development and progression of colon cancer. Medicinal plants like *Astragalus mongholicus* Bunge and *Curcuma aromatica* Salisb. (AC) have shown preferable therapeutic effect on cancer therapy, especially digestive tract tumors like colon cancer. However, the precise mechanisms of AC inhibiting colon cancer, particularly in relation to the gut microbiome and bile acid dynamics, are not fully understood.

**Methods:**

Our research aimed to investigate the anti-tumor properties of AC in mice with CT26 colon cancer and further investigate its underlying mechanism via intestinal microbiota. The size and pathological changes of solid tumors in colon cancer are used to evaluate the inhibitory effect of AC on colon cancer. Metagenomics and 16s rRNA gene sequencing were employed to clarify the dysbiosis in the gut microbiome of colon cancer and its impact on colon cancer. The levels of bile acids (BAs) in the feces of mice from each group were measured using UPLC-Qtrap-MS/MS.

**Results:**

AC effectively suppressed the growth of colon cancer and reduced histological damage. Notably, AC treatment led to changes in the gut microbiome composition, with a decrease in pathogenic species like *Citrobacter* and *Candidatus_Arthromitus*, and an increase in beneficial microbial populations including *Adlercreutzia*, *Lachnospiraceae*_UCG-001, and *Parvibacter*. Additionally, AC altered bile acid profiles, resulting in a significant decrease in pro-carcinogenic bile acids such as deoxycholic acid (DCA) and lithocholic acid (LCA), while increasing the concentration of the cancer-inhibitory bile acid, ursodeoxycholic acid (UDCA). Tracking and analyzing the data, AC may mainly upregulate FabG and baiA genes by increasing the relative abundance of *Adlercreutzia* and *Parvibacter* bacteria, which promoting the metabolism of pro-carcinogenic LCA.

**Discussion:**

These findings provide strong evidence supporting the role of AC in regulating gut microbiome-mediated bile acid metabolism, which is crucial in impeding the progression of colon cancer.

## Graphical abstract

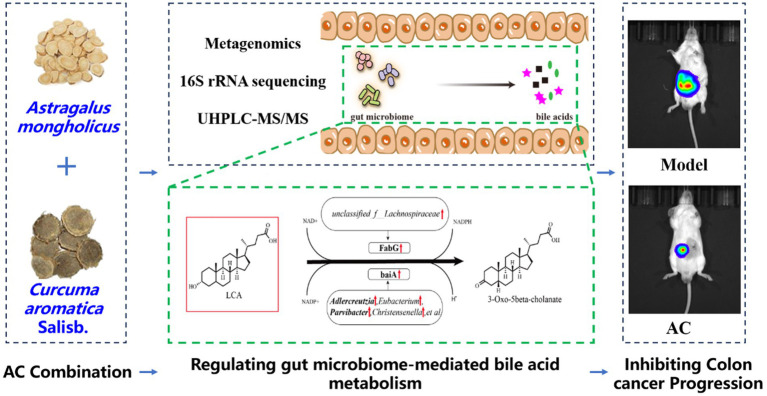

## 1 Introduction

Colon cancer (CC) is one of the most prevalent and lethal forms of cancer (Jia et al., 2022). Notably, there has been a significant rise in the incidence rate of colon cancer among young patients. By 2030, it is estimated that 10.9% of all colon cancers will be diagnosed in individuals under 50 years of age, compared to the current rate of 4.8% in 2010 (Bailey et al., 2015). The gut microbiota is known to play a crucial role in the development of various diseases, particularly gastrointestinal tract tumors. Studies have demonstrated that an increased abundance of *Eubacteria* and *Roseburia*, as well as a greater overall diversity in the microbiome, are associated with substantial inhibition of tumor growth in both subcutaneous and orthotopic colon cancer models (Zheng et al., 2020). Conversely, the enrichment of three common commensal bacteria, namely *Fusobacterium nucleatum*, *Bacteroides fragilis* and *Escherichia coli*, has been implicated in promoting the growth and progression of colon tumors (Leystra and Clapper, 2019). Furthermore, *Lactobacillus reuteri*, a beneficial microbe, has proven to be protective against colorectal cancer (Bell et al., 2022). Apart from the gut microbiota, bile acids, one of its primary metabolites, also play a significant role in cancer progression. Deoxycholate (DCA), a secondary bile acid, has been found to be markedly elevated in cases of multiple polypoid adenomas and intramucosal carcinomas. In contrast, ursodeoxycholic acid (UDCA), another secondary bile acid produced by *Clostridium* species, has demonstrated the ability to impede the occurrence of colon cancer (Khare et al., 2008).

Derived primarily from medicinal plants, Traditional Chinese Medicines (TCMs) have gained recognition for their therapeutic application in treating malignant tumors. They are valued for their multi-pathway, multi-target actions, minimal side effects, and potential to enhance immunity (Fan et al., 2020). One notable medicinal herb used in TCM is *Astragalus mongholicus* Bunge, which is renowned for its ability to tonify deficiencies. It exhibits anti-tumor properties by modulating immune responses, demonstrating anti-inflammatory and antioxidant activities, and directly inhibiting tumor proliferation (Auyeung et al., 2016). Another frequently used herb, *Curcuma aromatica* Salisb., is known for its ability to activate blood circulation. It contains active ingredients such as curcumin and β-elemene, which have been identified as regulators of multiple carcinogenic pathways (Lim et al., 2010; Tong et al., 2020). Preliminary studies suggest that the combination of *Astragalus mongholicus* Bunge and *Curcuma aromatica* Salisb. (AC) may provide anti-colorectal cancer benefits, primarily by modulating the gut microbiota (Gu et al., 2021), such as the abundance of *Lactobacillu* and *Mucispirillum* were significantly increased. And *Lactobacillus* was reported to reduce the content of intestinal BA via inhibiting the *de novo* synthesis of BA and increasing the excretion of BA through the intestinal FXR-FGF-15 signaling pathway. *Astragalus polysaccharides*, the main effective component of *Astragalus mongholicus* Bunge, significantly regulated most microorganisms, such as *Bifidobacterium pseudolongum*, *Lactobacillus johnsonii* and *Lactobacillus*, the changes in abundance of these microorganisms were related to the increase of metabolites, which could control tumor growth (Ding et al., 2021). The main active ingredients of *Curcuma aromatica* Salisb., curcumin showed the dose-dependent suppressive effect on inflammatory response and gut microbiota profile in high-fat fed C57BL/6 mice (Bertoncini-Silva et al., 2023). The intricate mechanisms supporting this therapeutic effect, however, still need to be clarified. Therefore, it is imperative to conduct a thorough investigation into the interaction between the drug pair and the intestinal microbiome. This investigation should primarily focus on determining their potential role in modifying carcinogenic pathways within the gastrointestinal tract.

This study implemented a comprehensive approach, utilizing Metagenomics and 16S rRNA sequencing to analyze the alterations in gene expression, gut microbiota, and bile acid profiles in a colon cancer mouse model. The primary objective of this investigation was to examine the influence of the AC combination on the gut microbiome, thereby affecting the metabolic pathways of bile acids and exerting suppressive effects on colorectal cancer progression. By doing so, this research aims to uncover fresh perspectives in the development of natural and effective anticancer therapeutics, while also identifying potential targets for the treatment of colorectal cancer using AC. Ultimately, this will expand the array of strategies and possibilities available for cancer therapy.

## 2 Materials and methods

### 2.1 Sample preparation

The water extract of *Astragalus mongholicus* Bunge and *Curcuma aromatica* Salisb. (referred to as AC) used in this study was prepared following established protocols (Gu et al., 2022). Briefly, 400 g of *Astragalus mongholicus* Bunge and 200 g of *Curcuma aromatica* Salisb. were weighed and subjected to two rounds of extraction using a heat reflux system. Each extraction involved 10 volumes of water and lasted for 60 min. The plants’ essential oil components were extracted using a Soxhlet extract unit. The resulting AC extract had a measured content of calycosin-7-O-glucoside, ononin, calycosin, quercetin, formononetin, bisdemethoxycurcumin, demethoxycurcumin, curcumin, curdione, soyasaponin I, astragaloside II, astragaloside IV, astragaloside I and germacrone at 4.701, 0.717, 0.296, 0.005, 0.578, 0.001, 0.003, 0.002, 2.999, 0.019, 0.112, 0.020, 0.289 and 0.459 mg/g (Supplementary Figure S1; Supplementary Table S1) via UPLC-MS/MS. The confirmation of the plant species, *Astragalus mongholicus* Bunge [Fabaceae] and *Curcuma aromatica* Salisb. [Zingiberaceae], was provided by Professor Tulin Lu of Nanjing University of Chinese Medicine.

### 2.2 Animal modelling and AC treatment

The mice used in this study were colon cancer models created through orthotopic transplantation tumor modeling, following a previously described method (Sun et al., 2021). Five breeder mice were subcutaneous in the right axilla injected with 1 × 10^6^ CT26-luc cells (BeNa Culture Collection) in RPMI-1640 medium supplemented with 10% fetal bovine serum, 100 U/mL penicillin, and 100 U/mL streptomycin. After approximately 5 days, when the tumor in the axilla reached the size of a soybean, the mice were humanely euthanized. The tumor was then removed and cut into small 1 mm^3^ cubes. Each mouse in the experimental group underwent a surgical procedure under isoflurane anesthesia (4% for induction; 1.5% for maintenance), in which a tumor cube was attached to the scratched caecum site in the lower left abdomen using histoacryl adhesive. The mice were then carefully sutured and treated with anti-infective agents. They were returned to their cages for postoperative care. The success rate of establishing the orthotopic-transplanted colon cancer model was 100%. In contrast, the sham group underwent all the same procedures as the experimental group, except for the tumor grafting.

A total of 20 mice with cancer were randomly allocated into the model group, and the AC group treated with AC extract at the dosage of 3 g/kg/day based on the clinical administration dose. Another 10 mice were considered as the sham group which mice also received surgical incisions in the left lower abdomen except for tumor grafting. For a duration of 14 days, 10 mice in each group received drug administration via gavage. In contrast, mice in the sham and model groups were administered normal saline at an equivalent dosage.

The study followed aseptic principles and complied with NIH guidelines for laboratory animal care. No post-surgery antibiotics were needed. Male BALB/c mice, aged 6–8 weeks and weighing 20 ± 2 g, were sourced from Huaxing Experimental Animal Farm (Zhengzhou, China). They were housed at Nanjing University of Chinese Medicine in a controlled environment (temperature: 26°C, humidity: 45%) under license SCXK (Zhe) 2019-0002, adhering to relevant regulations.

### 2.3 Live mouse bioluminescence imaging of tumor growth

One hour after the 10th day of drug administration, 200 μL D-Fluorescein Potassium Salt (40902ES08, Yisheng Biotechnology Co., Ltd., 15 mg/mL in PBS) were injected intraperitoneally to mice prior to imaging by a small animal live imaging device (IVIS Spectrum, PerkinElmer). After approximately 10 min, anesthetize mice in an induction chamber with 4% isoflurane/O_2_ at a flow rate of 2 L/min. Luminescent photographic images were obtain with low power X-ray, exposure to 30, field of view (FOV) to 25, object height to 1.5 cm. After acquiring initial images, adjust settings and obtain an optimized image.

### 2.4 Sample collection

Stool specimen collection: To collect stool specimens, the mice were first immobilized prior to anesthesia and euthanasia. Their tails were gently elevated and the lower abdomen was delicately massaged to facilitate defecation. Using sterile EP tubes, fresh feces were directly collected and promptly preserved in liquid nitrogen. Subsequently, the samples were transferred to a −80°C refrigerator for long-term storage.

Tissue sample collection: After performing cervical dislocation to humanely sacrifice the mice which are isoflurane anesthesia (4% for induction), an abdominal autopsy was conducted to obtain tissue samples of the colon and tumors. These collected tissue samples were then carefully preserved either in 4% paraformaldehyde or liquid nitrogen, depending on the subsequent processing requirements.

### 2.5 Histopathological studies

Hematoxylin–Eosin staining was conducted on 4% paraformaldehyde-fixed paraffin-embedded colon tissues and 3 mm solid tumor sections, following the manufacturer’s protocol (Leagene, DH0006). The resulting histological images were observed using an IX51 microscope manufactured by Olympus Corporation (Japan). The evaluation of colon tissues and solid tumors was performed blindly, adopting the specified method. Subsequently, pathobiological examinations were carried out and scored in a blinded manner.

### 2.6 Faecal metagenomic analysis

#### 2.6.1 DNA extraction

About 0.5 g fecal material was processed to extract genomic DNA by the PF Mag-Bind Stool DNA Kit (Omega Bio-tek, Norcross, GA, United States) in strict accordance with the manufacturer’s instructions. The extracted DNA was subsequently subjected to assessment of its concentration and purity, employing TBS-380 and NanoDrop2000, respectively. To evaluate the quality of the DNA, a 1% agarose gel electrophoresis was conducted.

#### 2.6.2 Metagenomic sequencing process

DNA samples were fragmented to 350 bp using the Covaris M220 instrument. Paired-end libraries were constructed using the NEXTFLEX Rapid DNA-Seq kit. Sequencing was performed on the Illumina Novaseq 6,000 platform at Majorbio Bio-Pharm Technology Co., Ltd., following the manufacturer’s instructions. The data is available in the NCBI Short Read Archive database under accession number SRP479162.

### 2.7 Faecal bile acids analysis

Feces were flash-frozen in liquid nitrogen and stored at-80°C. Bile acid standards were dissolved in methanol to create a 1 mg/mL stock solution. Fecal samples were carefully weighed (20 mg) and mixed with a methanol–water mixture (ratio of 4:1). Grinding and ultrasound treatment were performed to extract the compounds. The samples were then centrifuged and 200 μL of the supernatant was collected for machine detection.

The analysis of the sample was conducted using ultra performance liquid chromatography-triple quadrupole linear/ion trap of mass spectrometry (UPLC-Qtrap-MS/MS) on an ExionLC AD system coupled with a QTRAP® 6,500+ mass spectrometer (Sciex, United States) at Majorbio Bio-Pharm Technology Co. Ltd. (Shanghai, China). To separate the metabolites, a Waters BEH Amide column (150 × 2.1 mm, 1.7 μm) was employed and maintained at 40°C. The separation was achieved with a flow rate of 1 mL/min using a mobile phase consisting of two solvents: solvent A (95% acetonitrile in water with 0.4% formic acid and 20 mM ammonium formate) and solvent B (95% acetonitrile in water with 0.4% formic acid and 20 mM ammonium formate). The solvent gradient was programmed as follows: 0–1 min, 0–10% B; 1–2.6 min, 10–15% B; 2.6–3.5 min, 15–30% B; 3.5–4.0 min, held at 30% B; 4–4.1 min, 30 to 0% B; 4.1–6 min, held at 0% B. The samples were stored at 4°C throughout the analysis.

The linear range and standard curve were determined based on a plot of the concentration of the working standard solution (abscissa) against the corresponding peak area (ordinate). The obtained correlation coefficient (*r*) exceeded 0.99, indicating a robust linear relationship. The quantification limit was established using the signal-to-noise ratio method, wherein the signal obtained from a known low concentration sample was compared to that of a blank sample. Typically, the concentration corresponding to a signal-to-noise ratio of 10:1 (s/*N* = 10) was designated as the quantification limit. To ensure accuracy, each batch of samples was subjected to comprehensive testing involving a mixture of samples, blank samples, and standard samples. The recoveries of all 47 bile acids were determined by calculating the peak areas of the standards. Additionally, we prepared quality control (QC) samples by pooling an equal volume of 10 μL from each fecal sample to assess the stability of the UPLC-Qtrap-MS/MS system. It is noteworthy that the relative standard deviations (RSD%) for these QC samples were consistently below 15% (Supplementary Table S2).

### 2.8 16S rRNA gene sequencing analysis

Total microbial genomic DNA was extracted from cecum samples using the PF Mag-Bind Stool DNA Kit (Omega Bio-tek, Georgia, United States), following the manufacturer’s instructions. Following extraction, the quality and concentration of the DNA were assessed through 1.0% agarose gel electrophoresis and NanoDrop® ND-2000 spectrophotometry (Thermo Scientific Inc., United States), respectively. The extracted DNA was stored at-80°C until further use. For amplification of the V3-V4 hyper-variable regions of the bacterial 16S rRNA gene, the ABI GeneAmp® 9,700 PCR thermocycler (ABI, CA, USA) was employed. Each sample was subjected to triplicate amplification under cycling conditions. The resulting PCR product was purified from a 2% agarose gel, pooled in equimolar amounts, and subsequently subjected to paired-end sequencing on an Illumina PE300/PE250 platform (Illumina, San Diego, United States). Majorbio Bio-Pharm Technology Co. Ltd. (Shanghai, China) conducted the sequencing according to standard protocols. The raw sequencing reads were deposited into the NCBI Sequence Read Archive (SRA) database under the Accession Number: SRP479110.

We initially demultiplexed the raw FASTQ files with our in-house Perl script. Subsequently, fastp version 0.19.6 was used for quality filtering, followed by merging using FLASH version 1.2.11. The resulting optimized sequences were then clustered into operational taxonomic units (OTUs) at a 97% sequence similarity level, employing UPARSE 11. Taxonomy assignment for each representative sequence of the OTUs was performed using the RDP Classifier version 2.13 with a confidence threshold of 0.7 and the 16S rRNA gene database. We conducted metagenomic function prediction based on the representative sequences of the OTUs using PICRUSt2. For the calculation of metrics such as observed OTUs, Shannon index, Simpson index, and Chao1 index, we utilized the alpha_diversity.py script, which computed the rarefaction of OTUs. Additionally, to visualize the beta diversity, we employed the beta_diversity_through_plots.py script.

### 2.9 Statistical analysis

Data are expressed as mean ± SEM. Before testing for significant differences between the two groups, tests of normality (Shapiro–Wilk). Student’s *t*-test (parametric) or One-way ANOVA was used when the conditions of normality. When normality test failed, the Kruskal–Wallis test (non-para metric) was used. *p* < 0.05 and *p* < 0.01 were considered as statistically significant. All statistical analyses were performed using SPSS 27.0.

## 3 Results

### 3.1 AC suppresses colon cancer in tumor-bearing mice

**Figure 1:**
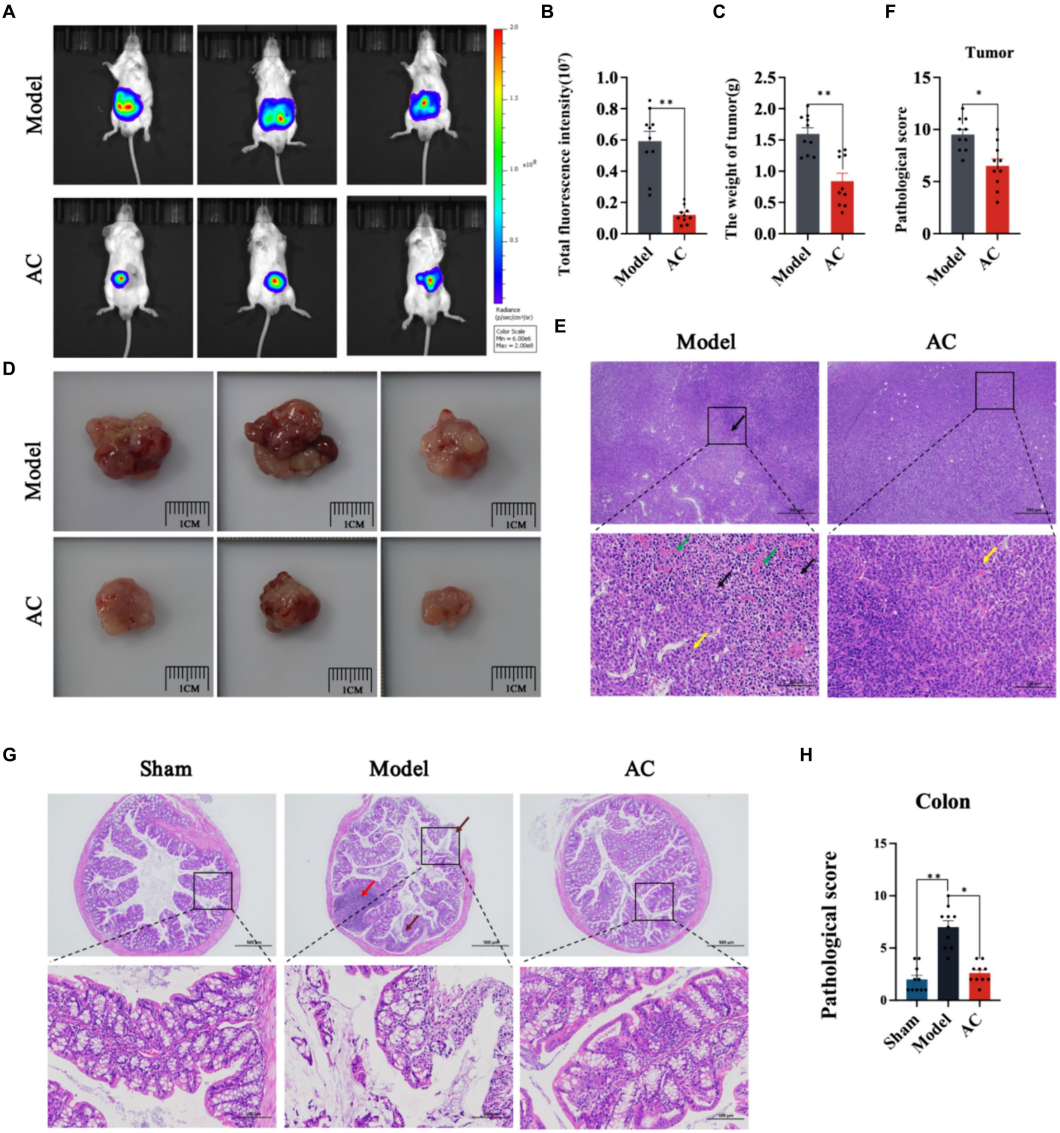
The effect of AC on inhibiting tumor growth CC tumor-bearing Mice. **(A)** Bioluminescent imaging of tumor growth 10th days following the orthotopic transplantation tumour model is shown. **(B)** Total fluorescence intensity of mice after AC administration. **(C)** The weight of tumor of mice after AC administration. **(D)** Endpoint tumor size in mice. **(E)** Pathological changes of tumor tissue [haematoxylin and eosin (HE) stain]. Occasional nuclear mitotic figures (yellow arrows). Eosinophilic amorphous material (black arrows). Vessels displayed congestion (green arrows). **(F)** Pathological score of tumor tissue. **(G)** Pathological changes of colon tissue (haematoxylin and eosin (HE) stain). Goblet cells (brown arrows), Lymphoid tissue in the submucosal layer (red arrows). **(H)** Pathological score of colon tissue (^**^p < 0.01, ^*^p < 0.05, *n* = 10, Student’s *t*-test).

In this study, we utilized *in vivo* bioluminescence imaging technology to noninvasively monitor tumor growth dynamics and assess the inhibitory effect of AC in tumor-bearing mice. Our findings demonstrated a significant decrease in fluorescence intensity upon AC administration at the tumor site (Figures 1A,B). Furthermore, as illustrated in Figures 1C,D, AC effectively suppressed both colon tumor volume and weight.

Pathological changes in tumor and colon tissue were assessed through HE staining. The model group in Figure 1E exhibited localized and irregular arrangements of tumor cells. These cells displayed nuclear pleomorphism and a high nuclear-cytoplasmic ratio, with occasional observation of nuclear mitotic figures (indicated by yellow arrows). Moreover, areas of tumor cell sheet-like necrosis with fragmented nuclei were observed, along with the presence of eosinophilic amorphous material (indicated by black arrows). Locally, there was a noticeable increase in proliferative blood vessels, some of which exhibited congestion (indicated by green arrows). The colonic tissue of model mice demonstrated multiple erosions, loss of mucosal epithelium and glandular structures, thinning of the mucosal layer, reduced goblet cell (indicated by brown arrows), and extensive lymphoid tissue in the submucosal layer (indicated by red arrows) (Figure 1G). Subsequent to AC treatment, significant improvements in the pathological changes of tumor and colon tissue were observed, as indicated by the reduced pathological scores (Figures 1F,H).

### 3.2 AC improves the intestinal microbiota mediated bile acid pathway of feces in tumor-bearing mice

**Figure 2:**
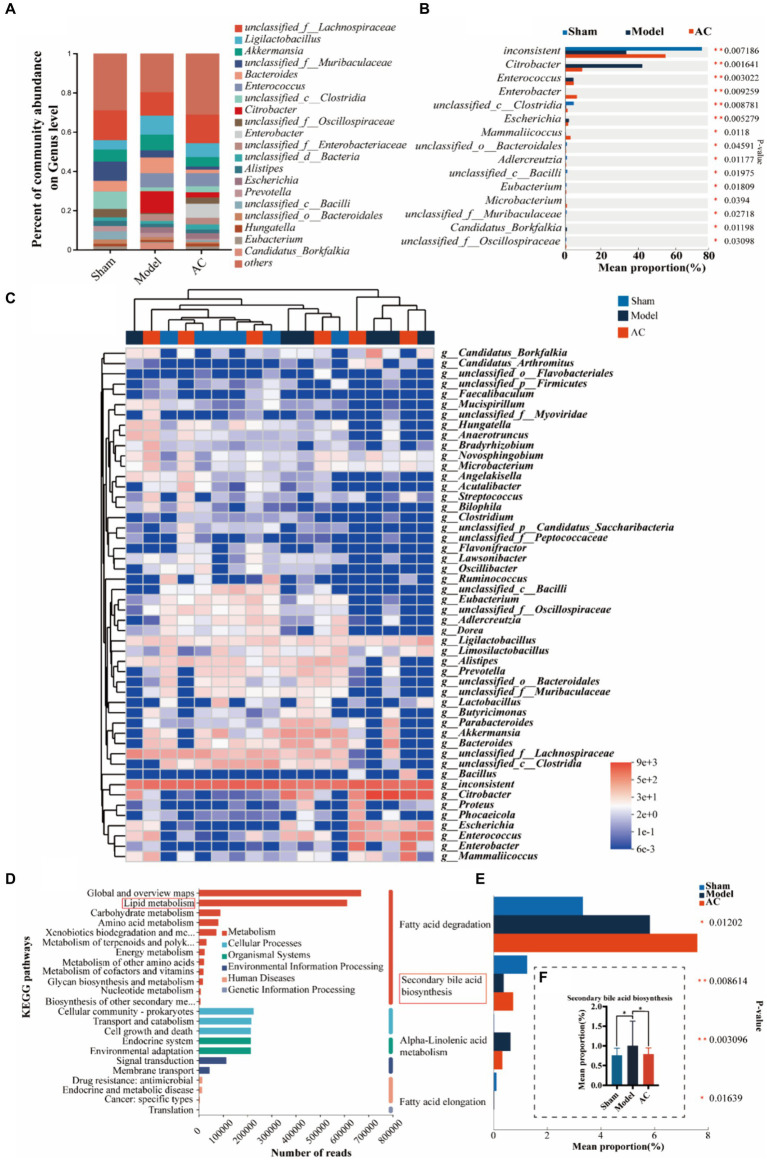
The effect of AC on the intestinal microbiota-mediated bile acid pathway of feces in tumor-bearing Mice. **(A)** Percent of gut microbial community abundance on Genus level. **(B)** Kruskal–Wallis *H* test bar plot of gut microbial community on Genus level. **(C)** Heatmap analyses of gut microbiota at the genus level in top 50. **(D)** KEGG enrichment analysis for differentially expressed genes on pathway level 2. **(E)** Kruskal–Wallis *H* test bar plot of Lipid metabolism pathway. **(F)** Kruskal–Wallis *H* test bar plot of Secondary bile acid biosynthesis (^**^p < 0.01, ^*^p < 0.05, *n* = 6, Kruskal–Wallis test).

To investigate dysbiosis in the gut microbiome of colon cancer mice and its impact on colon cancer, a metagenome-wide association study was conducted. The analysis focused on the genus level of the gut microbiota, as illustrated in Figure 2A. The main genera identified were *unclassified_f__Lachnospiraceae* and *Ligilactobacillus*. When comparing the microbiota composition of mice with tumors to that of sham mice, significant changes were observed. Specifically, there was an increase in the relative abundances of *Ligilactobacillus* and *Akkermansia*, whereas *unclassified_f__Lachnospiraceae* and *unclassified_f__Muribaculaceae* decreased. Interestingly, administration of AC resulted in a reversal of the altered microbiota composition at the genus level. Further analysis revealed significant differences in the composition and abundances of intestinal flora between the model and sham groups, with *Citrobacter* and *Escherichia* showing notable increases, while *unclassified_c_Clostridia* and *unclassified_o_Bacteroidales* were significantly reduced (*p* < 0.01, *p* < 0.05, Figure 2B). Importantly, the treatment with AC induced significant alterations in the microbiota structure, as compared to the model group (Figures 2A–C). This underscores the potential impact of AC on the gut microbiome in the context of colorectal cancer.

Out of a total of 1.6 million genes, we identified 4,471 differential genes that exhibited a significant association with disease status (*p* < 0.01, determined by Wilcoxon rank-sum test). To investigate potential microbial-derived pathways associated with colon cancer, we conducted Kyoto Encyclopedia of Genes and Genomes (KEGG) enrichment analysis on these 4,471 genes. Our analysis revealed that lipid metabolism was the second most prominently affected metabolic pathway (Figure 2D), implying its potential involvement in CC pathogenesis and the interplay between intestinal microbiota and colon cancer. Further exploration of the genes within the lipid metabolism pathway unveiled the significant ranking of the secondary bile acid biosynthesis pathway (Figure 2E). Notably, as demonstrated in Figure 2F, the model group exhibited a substantial increase in the mean proportion of the secondary bile acid biosynthesis pathway compared to the sham group, which was subsequently attenuated upon AC treatment.

### 3.3 AC modulated the faecal bile acids in tumor-bearing mice

**Figure 3:**
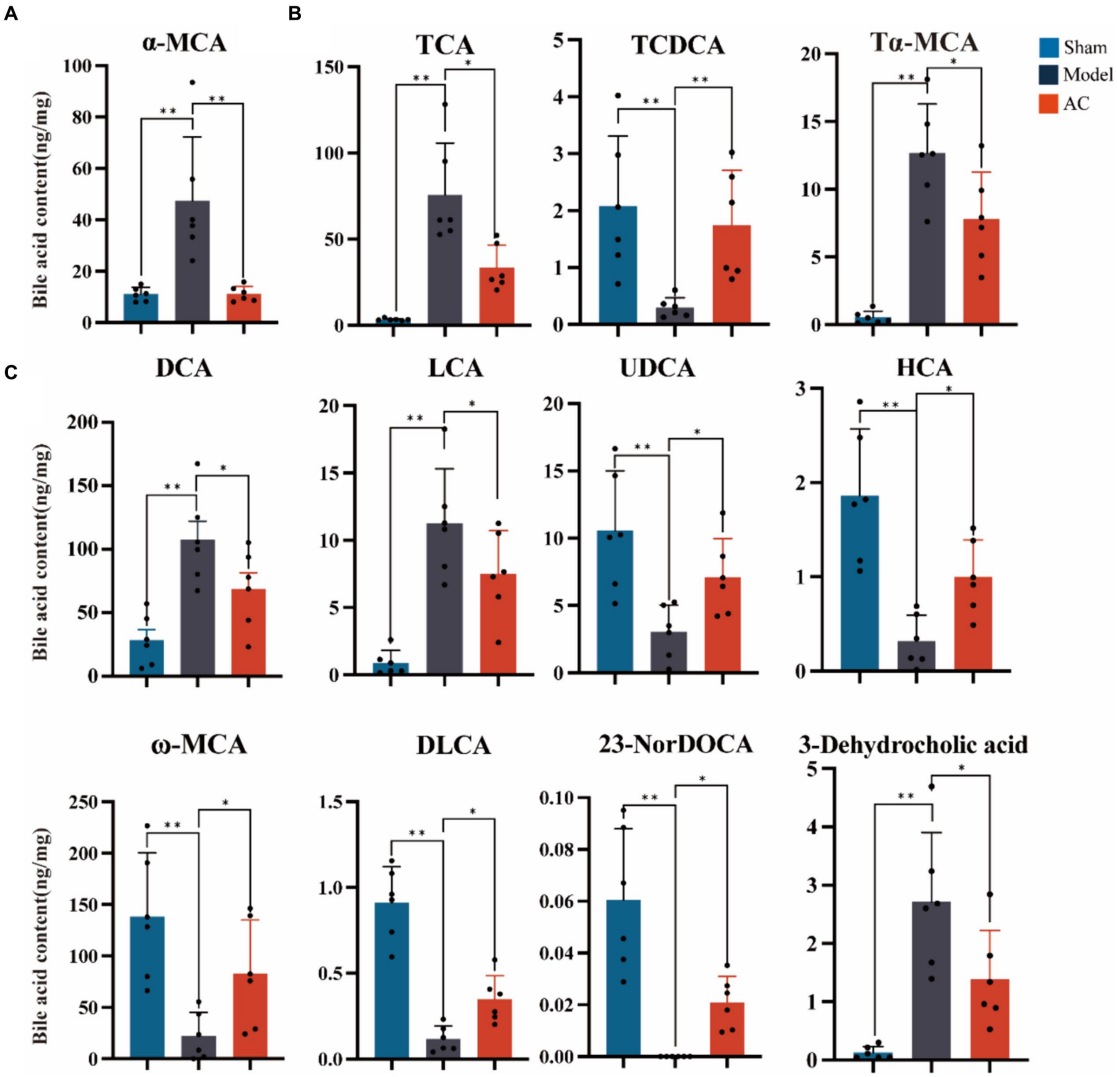
The effect of AC on faecal bile acids in tumor-bearing Mice. **(A)** Representative differentiated primary bile acid. **(B)** Representative differentiated conjugated bile acids. **(C)** Representative differentiated secondary bile acids (^**^p < 0.01, ^*^p < 0.05. *n* = 6, One-way ANOVA).

The secondary bile acid biosynthesis pathway, regulated by AC administration, is considered the primary metabolic pathway in CC. The levels of bile acids (BAs) in the feces of mice from each group were measured using UPLC-Qtrap-MS/MS Through this method, a total of 47 BAs were successfully separated and demonstrated varying content among the different groups (Supplementary Figure S2). In Figure 3A, it is evident that tumor-bearing mice exhibited significantly higher levels of the primary BA alpha-muricholic acid (α-MCA) compared to the sham group. However, after the administration of AC, these elevated levels were dramatically reversed (*p* < 0.01). Regarding the conjugated BAs, the levels of taurocholic acid (TCA) and tauro-α-muricholic acid (Tα-MCA) were found to be significantly higher in the model group than in the sham group, while the concentration of taurochenodeoxycholic acid (TCDCA) noticeably decreased (*p* < 0.01, Figure 3B). Remarkably, the administration of AC effectively modulated the levels of these bile acids in tumor-bearing mice, causing them to approach levels comparable to those seen in the sham group (*p* < 0.01, *p* < 0.05).

The results shown in Figure 3C indicate a noteworthy increase in the levels of secondary bile acids, specifically deoxycholic acid (DCA), lithocholic acid (LCA), and 3-dehydrocholic acid, in the model group compared to the sham group (*p* < 0.01). In contrast, the model group demonstrated a significant decrease in the concentrations of ursodeoxycholic acid (UDCA), hyocholic acid (HCA), omega-murichoclic acid (ω-MCA), dehydrolithocholic acid (DLCA), and 23-Nordeoxycholic acid (23-NorDOCA) (*p* < 0.01). However, the administration of AC resulted in a significant reversal of these alterations in bile acid levels in tumor-bearing mice (*p* < 0.05, *p* < 0.01).

### 3.4 AC ameliorated the diversity and abundance of bacterial communities in intestinal microbiota of tumor-bearing mice

**Figure 4:**
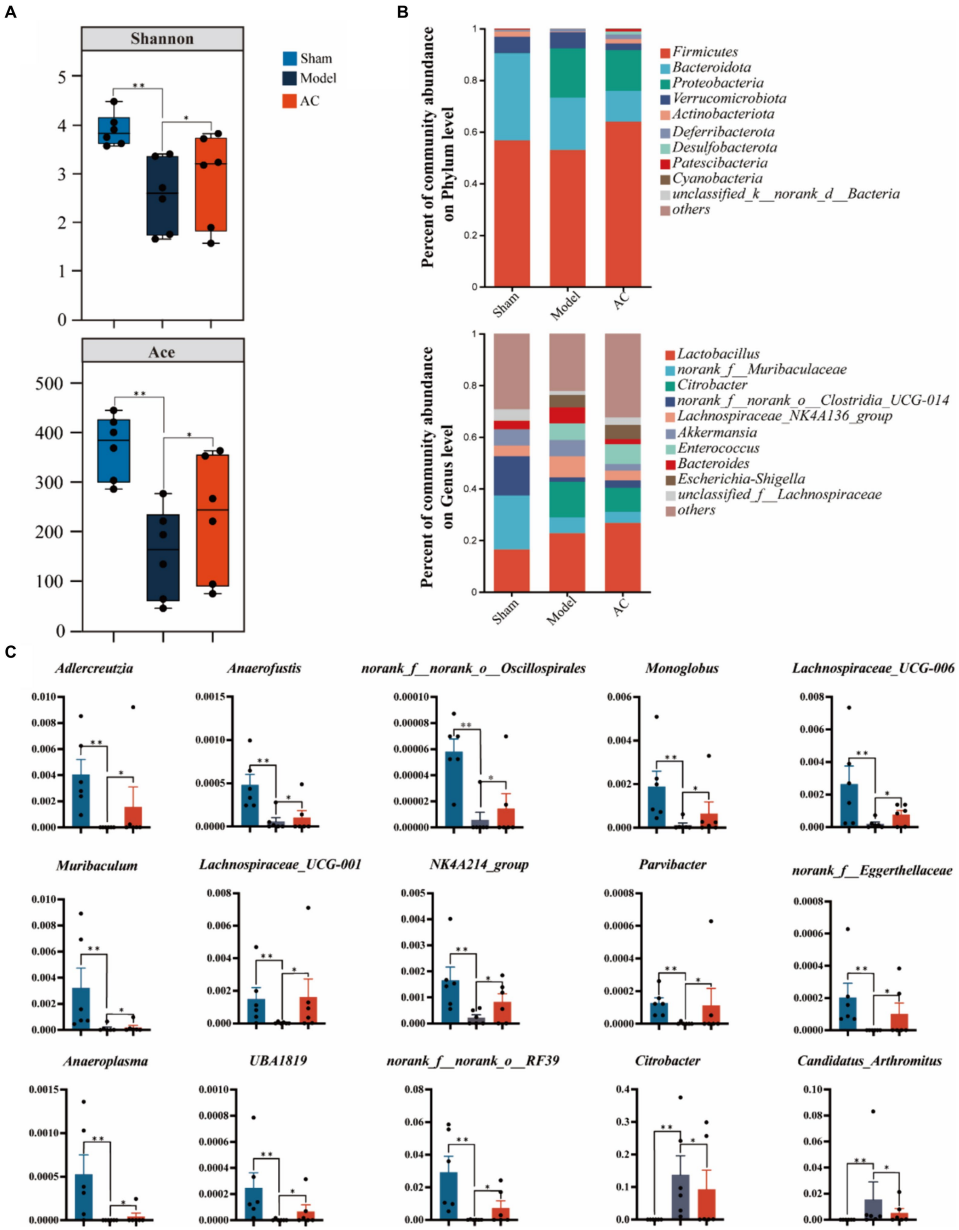
The effect of AC on the diversity and abundance of bacterial communities in intestinal microbiota of tumor-bearing Mice. **(A)** α-Diversity was measured by shannon and ace indexes. **(B)** Community bar plot analysis of gut microbiota at the phylum level and genus level. **(C)** Representative differentiated genera (^**^p < 0.01, ^*^p < 0.05, *n* = 6, Kruskal–Wallis test).

To examine the bacterial composition and abundance in the fecal samples of mice with colon cancer, we employed 16S rRNA sequencing. Analysis using the Shannon and Ace indices revealed that the alpha diversity of the mouse feces was lower in the colon cancer group compared to the sham group. This observation strongly suggests a decrease in both the abundance and diversity of the gut microbiota following colon cancer modeling. Interestingly, intervention treatment with AC led to varying degrees of improvement in the intestinal microbial diversity and richness among the colon cancer mice, as depicted in Figure 4A.

As shown in Figure 4B, we can observe a distinct stratification of the gut microbiota at both the phylum and genus levels. At the phylum level, it is evident that *Firmicutes* and *Bacteroidota* are the dominant microbial taxa. However, in the Model group, there is a noticeable shift, characterized by an increase in *Proteobacteria* and a decrease in the previously dominant *Firmicutes* and *Bacteroidota*. This shift indicates a disruption of the microbial equilibrium, referred to as dysbiosis. Furthermore, this dysbiosis is also represented by variations in less abundant phyla such as *Verrucomicrobiota* and *Actinobacteriota*. At the genus level, the analysis of the community barplot illustrates the distribution of gut microbiota among three groups: Sham, Model, and AC treatment. In the Model group, there is a significant alteration in the microbial composition, with an increased abundance of *Citrobacter* and *Lactobacillus*, along with a decrease in *norank_f__Muribaculaceae* and *norank_f__norank_o__Clostridia_UCG-014*. These changes indicate a modification in the gut microbiome due to the modeled condition. Furthermore, the barplot following AC treatment reveals a modification in the structure of gut microbiota, resembling that observed in the Model group. This suggests that the AC treatment has a restorative influence on the gut microbiota.

Out of the 49 genera that exhibited differential abundance (Supplementary Table S3), a notable increase was observed in the abundances of *Citrobacter* and *Candidatus_Arthromitus*. On the contrary, a significant decrease in abundance was noted for *Adlercreutzia*, *Anaerofustis*, *norank_f__norank_o__Oscillospirales*, *Lachnospiraceae*_*UCG-006*, *Muribaculum*, *Lachnospiraceae*_*UCG-001*, *NK4A214_group*, *Parvibacter*, *norank_f__Eggerthellaceae*, *Anaeroplasma*, *UBA1819*, and *norank_f__norank_o__RF39* (*p* < 0.01, *p* < 0.05, as indicated in Figure 4C). These findings highlight the significant changes in the microbial composition.

### 3.5 AC alternated intestinal microbiota genes associated with CC

**Figure 5:**
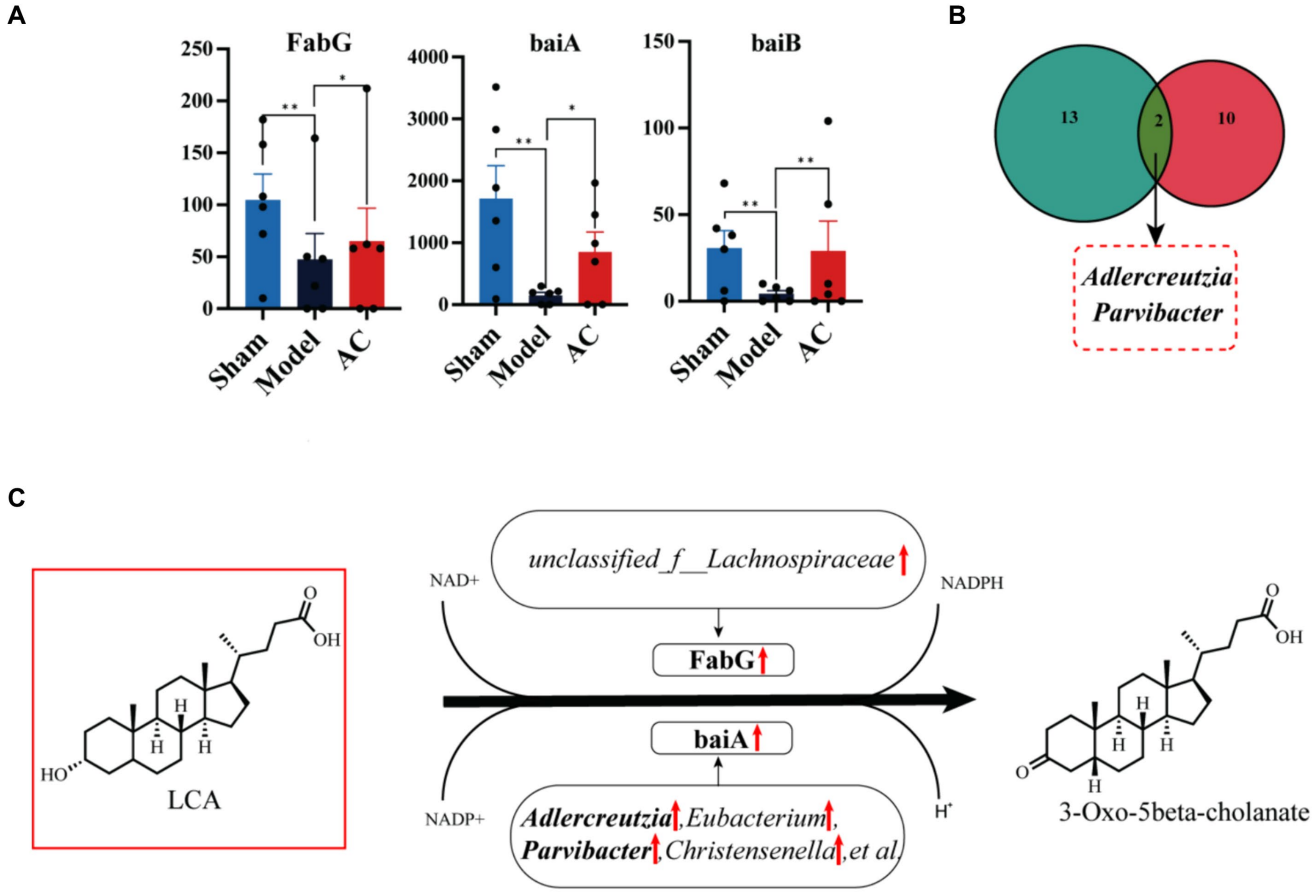
The effect of AC on the intestinal microbiota genes of tumor-bearing Mice. **(A)** Representative differentiated genes in 15 genes involved in the secondary bile acid biosynthesis pathway. **(B)** The venn diagram analysis of representative differentiated genera in 16 s rRNA sequencing with annotated bacteria in the Nr database. **(C)** The influence of gut microbiota community genes on LCA biochemical reactions (^**^p < 0.01, ^*^p < 0.05, *n* = 6, Kruskal–Wallis test).

Next, we compared the expression levels of 15 genes involved in the secondary bile acid biosynthesis pathway among three groups: Sham, Model, and AC. Supplementary Table S4 provides a detailed comparison. Among these genes, FabG, baiA, and baiB showed varying abundance in the three groups. Figure 5A clearly demonstrates that the expression levels of baiB, FabG, and baiA were significantly lower in the Model group compared to the Sham group. However, after the administration of AC, these expression levels normalized (*p* < 0.01, *p* < 0.05). Analysis of the Nr database revealed that these three genes are closely associated with 33 different microorganisms, and statistically significant differences were observed in 12 of these species. Then the bacterial species *Adlercreutzia* and *Parvibacter* were obtained by Venn diagram analysis of 12 microorganisms and 15 differential *bacteria* analyzed by 16S rRNA sequencing (Figure 5B). Considering the analysis of bile acid variations, it is evident that the genes FabG and baiA are crucial in the secondary bile acid pathway, particularly in relation to AC treatment. Figure 5C illustrates the metabolic conversion of lithocholic acid (LCA) to 3-oxo-5beta-cholanate within the secondary bile acid biosynthesis pathway. This conversion involves a series of enzymatic reactions facilitated by specific genes and associated bacterial genera. The diagram details the upregulation of the FabG gene within the *unclassified_f*__*Lachnospiraceae* genus. This indicates that this bacterial gene is likely involved in the metabolism of LCA. Moreover, there is a significant correlation between the upregulated expression of the baiA gene and the increased abundance of bacterial genera such as *Adlercreutzia*, *Eubacterium*, *Parvibacter*, and *Christensenella*, among others. This enhanced gene expression is closely associated with the metabolic conversion of lithocholic acid (LCA) to 3-oxo-5beta-cholanate. Finally, the upregulation of baiA and baiB suggests their pivotal role in the secondary bile acid pathway.

## 4 Discussion

Colon cancer is a globally prevalent gastrointestinal malignancy marked by increasing incidence and mortality rates. Clinical treatment methods for this disease primarily consist of surgery, chemotherapy, or a combination thereof, depending on disease progression (Miller et al., 2016). Unfortunately, these therapeutic approaches often give rise to a range of side effects, including intestinal dysfunction, bladder dysfunction, and sexual dysfunction. Consequently, there is a pressing need to explore and develop natural and potent antineoplastic agents that offer considerable efficacy while minimizing toxicity and adverse reactions. This pursuit has become a critical focal point in the realm of antitumor drug research and development.

Traditional Chinese Medicine (TCM) has been proven to enhance the effectiveness of chemotherapy, radiotherapy, targeted therapy, and immunotherapy, thus reducing the damage caused by these treatments. This not only supports cancer patients in their battle against the disease but also improves their overall quality of life (Wang et al., 2020). *Astragalus mongholicus* Bunge is commonly utilized in TCM for the treatment of various inflammatory diseases and cancers. Its anti-tumorigenic properties in colorectal cancer (CRC) have been demonstrated. For instance, *Astragalus membranaceus* saponins have exhibited anticancer activity by modulating the PI3K/Akt/mTOR and ERK signaling pathways in colorectal cancer cells HCT116 and HT-29. In addition, *Astragalus polysaccharides* have demonstrated outstanding anti-tumor activities by inhibiting cell proliferation, promoting apoptosis, and regulating the immune response. Another TCM herb, *Curcuma aromatica* Salisb., contains bioactive substances such as curcumin and curcumol, which have shown effectiveness in cancer treatment. Curcuma exerts anti-tumor effects by modulating various components of the signaling cascades involved in cancer cell proliferation, invasion, and apoptosis processes (Tiwari and Jain, 2020). In a previous research study, we observed that the combination of *Astragalus membranaceus* and *Curcuma aromatica* Salisb. strengthened anti-colorectal cancer efficacy and effectively improved the diversity and richness of the intestinal microbiota (Sun et al., 2021). The oral bioavailability of astragaloside in *Astragalus mongholicus* Bunge poses significant challenges in oral bioavailability. We identified the components of the AC that entered the bloodstream, and the results showed that most of the Astragalus saponins and flavonoids from *Astragalus mongholicus* Bunge, as well as some of the volatile oil components and curcumins from *Curcuma Aromatica* Salisb. could not exert regulatory effects on the blood transfusion (Liu et al., 2022). The therapeutic value of *astragalus polysaccharides* can be explained from the perspective of “intestinal efficacy” (Liu et al., 2019). Curcumin has a low blood concentration after oral administration, but exists at a high concentration in the gastrointestinal tract. It interacts with the gut microbiota in the intestine to restore abnormal gut microbiota and provide treating benefits (Di Meo et al., 2019). In the present experiment, we conducted further investigations to explore the mechanism of action by which this combination improves the intestinal microbiota in the context of colon cancer.

Integrated metagenomics is employed to investigate alterations in microbial communities within colorectal cancer patients and predict the functional potential of these microbiomes. The gut microbiota of individuals with colon cancer is studied using 16S rRNA sequencing (Yang et al., 2019). The imbalance of gut microbiota was associated with the occurrence of colon cancer (Cheng et al., 2020), the increase of pathogenic bacteria enhanced a series of carcinogenic signaling pathways like WNT-β-catenin, MAPK and PI3K-AKT (Rubinstein et al., 2019). And probiotics could delay the development of tumors by reversing the imbalance of intestinal flora caused by the enrichment of pathogenic bacteria (Li et al., 2021; Owens et al., 2021). The gut microbiota plays a role in the metabolism of bile acids (BA), which in turn influences the occurrence and development of cancer. Approximately 5–10% of BAs are secreted into the colon, where they are biotransformed by the gut microbiota or excreted in feces. At the primary bile acid level, the increased α-MCA metabolism promoted cancer metastasis via affecting the recruitment of NKT cells to form a pre-metastatic niche in the liver (Cheng et al., 2023). At the level of conjugated bile acids, TCA and Tα-MCA acted as tumor promoters due to the gene toxin and FXR inhibition (Ridlon et al., 2016; Shang et al., 2022). While TCDCA can inhibit the proliferation and invasion of gastric cancer and induce its apoptosis (Shen et al., 2022). In secondary bile acids, DCA and LCA have been shown to have cytotoxic and carcinogenic properties, the increased DCA and LCA in the colon lead to DNA oxidative damage and increased mitotic activity, leading to intestinal cell carcinogenesis, reducing the content of both exerting anti-tumor effects (Shang et al., 2022). The abnormal increase of DCA and LCA also destroyed the intestinal mucosal barrier and induced intestinal inflammatory response to provide a microenvironment for cancer development and promotes the development of intestinal tumors (Bu et al., 2023). Elevated levels of DCA and LCA in the colon activate cellular mechanisms, including the Wnt/β-catenin and NF-κB signaling pathways, leading to DNA oxidative damage and increased mitotic activity (Jia et al., 2018). LCA, a potential initiator of tumorigenesis, functions by inducing cell cycle arrest and apoptosis through the generation of intracellular reactive oxygen species and fragmentation of genomic DNA. This DNA damage promotes frequent turnover of the colon epithelium and creates favorable conditions for the initiation and progression of cancer. Dietary UDCA supplementation, a natural agonist of TGR5, can significantly reduce the incidence of colon cancer through reducing the inflammatory response of colonic mucosa and damage of oxidative stress to cells (Guo et al., 2023; He et al., 2023). Nevertheless, 23-NorDOCA promotes cell proliferation, migration and invasion of hepatocellular carcinoma (Zuo et al., 2023). In our study, KEGG functional analysis revealed a close association between dysregulation of gut microbiome function and disturbances in the secondary bile acid metabolic pathway after AC administration. The results of 16S rRNA sequencing indicated an increase in richness and diversity of gut microbial communities following AC treatment. Specifically, AC intervention in tumor-bearing mice significantly upregulated beneficial bacteria such as *Adlercreutzia* and *Parvibacter*, while reducing harmful bacteria like *Citrobacter* and *Candidatus_Arthromitus*. Furthermore, the gut microbiota-mediated bile acid metabolism exhibited corresponding adjustments, including a decrease in carcinogenic DCA and LCA levels and an increase in cancer-inhibiting UDCA.

The gut microbiota contains the functional bile salt hydrolase (BSH), commonly found in key gut microbial groups such as *Lactobacilli*, *Bifidobacteria*, *Clostridium*, and *Bacteroides* (Wahlström et al., 2016). Secondary bile acids like DCA, LCA, and UDCA are synthesized from primary bile acids by removing the 7α/β-hydroxy group by BSH hydrolyzing. Several bile acid-inducible (bai) genes (Funabashi et al., 2020) are responsible for the 7α/β-dehydroxylation process, which is regulated by various strains. This underscores the significant role of these bile acid metabolites in colorectal cancer. Our research employed advanced metagenomic techniques to identify significant variations in two key genes, FabG and baiA, among the 15 crucial genes associated with the secondary bile acid metabolic pathway. The upregulation of FabG and baiA contributes to the conversion of LCA, leading to a reduction in its concentration within the host. Moreover, through the NR species annotation of these genes, we gained valuable insights into their microbial associations. Specifically, FabG exhibited a positive correlation with *unclassified_f*_*_Lachnospiraceae*, while *Adlercreutzia*, *Eubacterium*, *Parvibacter*, and *Christensenella* showed a similar relationship with baiA. This emphasizes the complex interplay between the expression of gut microbiota genes and host bile acid metabolism, enhancing our understanding of the functional diversity of the microbiota and its impact on colon cancer.

## 5 Conclusion

In summary, our study provides evidence that AC significantly impacts gut microbiota, preventing colon cancer. It modulates microbial composition, leading to protective bile acid secretion. AC may mainly upregulate FabG and baiA genes by increasing the relative abundance of *Adlercreutzia* and *Parvibacter* bacteria, thus promoting the metabolism of pro-carcinogenic LCA in mice with colon cancer. These insights can guide future strategies for using AC in colon cancer prevention.

## Data availability statement

The datasets presented in this study can be found in online repositories. The names of the repository/repositories and accession number(s) can be found in the article/Supplementary material.

## Ethics statement

The animal study was approved by Nanjing University of Chinese Medicine. The study was conducted in accordance with the local legislation and institutional requirements.

## Author contributions

XW: Data curation, Formal analysis, Investigation, Methodology, Writing – original draft. BZ: Data curation, Formal analysis, Methodology, Writing – original draft. YH: Methodology, Validation, Writing – original draft. RS: Formal analysis, Funding acquisition, Writing – original draft. XT: Investigation, Methodology, Writing – original draft. XC: Funding acquisition, Investigation, Writing – original draft. DT: Funding acquisition, Project administration, Writing – review & editing. JG: Data curation, Funding acquisition, Investigation, Resources, Writing – review & editing.
