# Supplementary figures and images for: Astragalus mongholicus Bunge and Curcuma aromatica Salisb. modulate gut microbiome and bile acid metabolism to inhibit colon cancer progression
Source: Front Microbiol. 2024 Jun 17;15:1395634. doi: 10.3389/fmicb.2024.1395634 (PMC11215047; doi:10.3389/fmicb.2024.1395634)

# Supplementar FIGURE S1

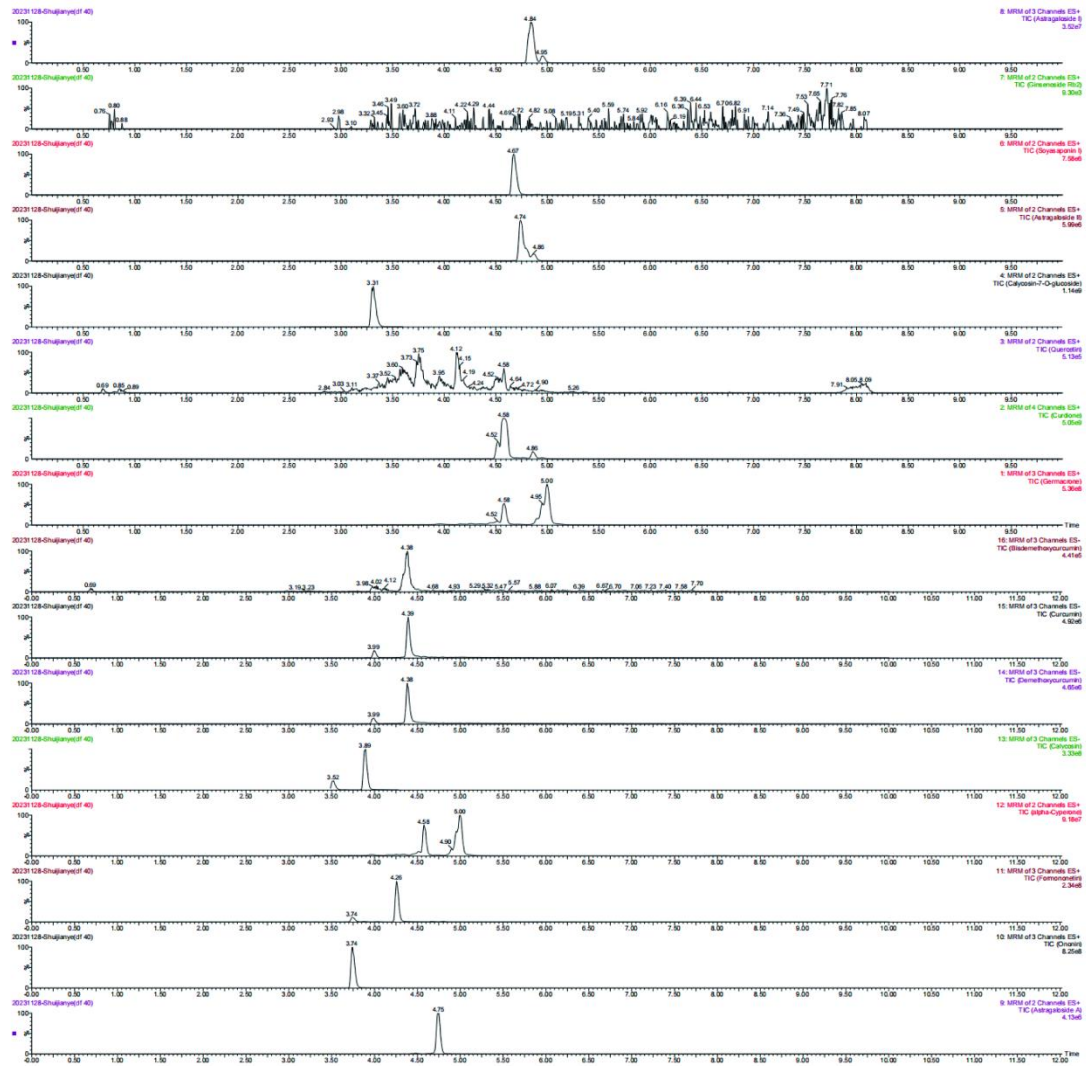

## Supplementary FIGURE S2

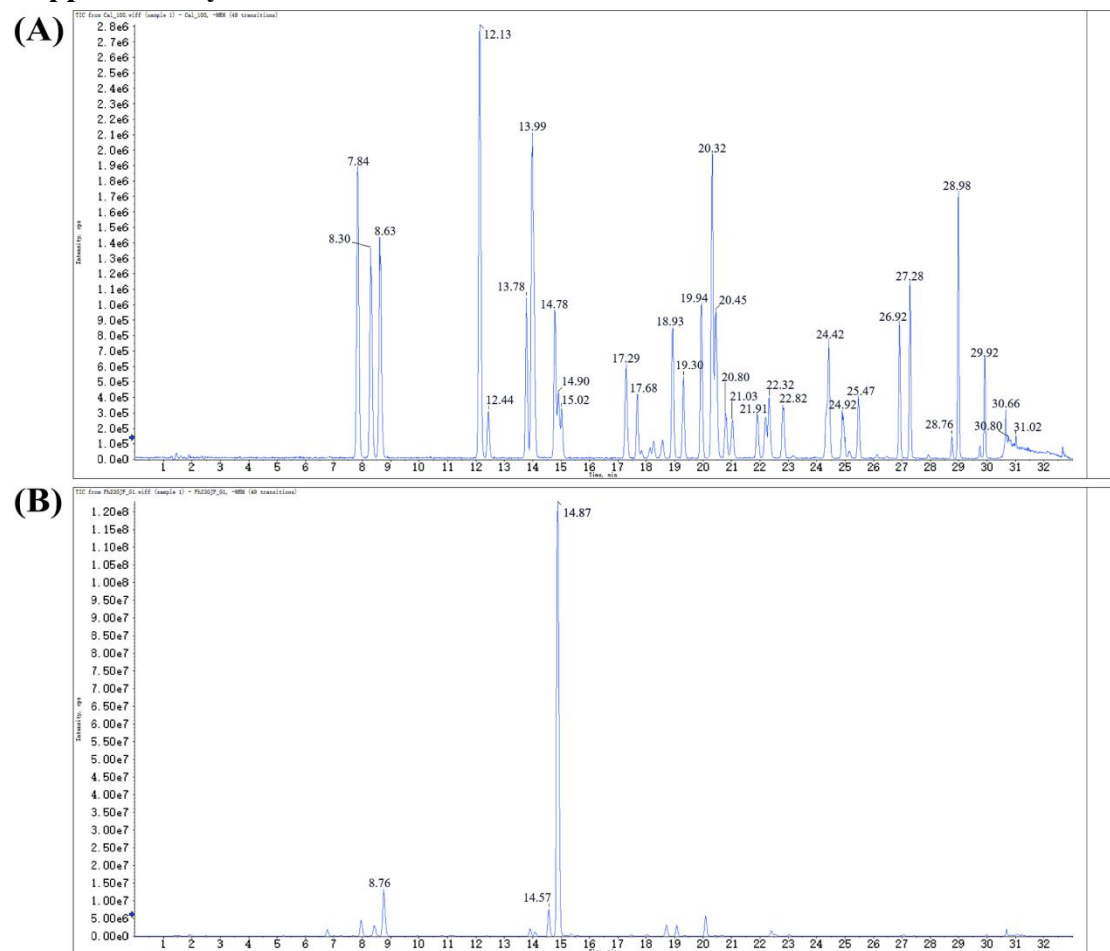

Supplement: Supplementary file 1 [file Image_1.pdf]
