# Supplementary material for: Astragalus mongholicus Bunge and Curcuma aromatica Salisb. modulate gut microbiome and bile acid metabolism to inhibit colon cancer progression
Source: Front Microbiol. 2024 Jun 17;15:1395634. doi: 10.3389/fmicb.2024.1395634 (PMC11215047; doi:10.3389/fmicb.2024.1395634)
Supplement: Supplementary file 2 [file Table_1.docx]

**Supplementary Table S1 Determination results of the main components of AC via UPLC-MS/MS**

| **Main components** | **Rt (min)** | **Precursor** | **Product** | **ESI(-/+)** | **Content(mg/g)** |
| --- | --- | --- | --- | --- | --- |
| Calycosin-7-O-glucoside | 3.51 | 447.0 | 285.0 | + | 4.701 |
| Ononin | 3.90 | 431.0 | 269.0 | + | 0.717 |
| Calycosin | 4.09 | 283.0 | 268.0 | - | 0.296 |
| Quercetin | 4.11 | 303.0 | 153.0 | + | 0.005 |
| Formononetin | 4.44 | 269.2 | 197.0 | + | 0.578 |
| Bisdemethoxycurcumin | 4.57 | 307.0 | 186.5 | - | 0.001 |
| Demethoxycurcumin | 4.59 | 337.0 | 119.0 | - | 0.003 |
| Curcumin | 4.61 | 367.0 | 217.0 | - | 0.002 |
| Curdione | 4.78 | 237.0 | 135.0 | + | 2.999 |
| Soyasaponin I | 4.81 | 965.5 | 331.0 | + | 0.019 |
| Astragaloside II | 4.93 | 849.5 | 669.5 | + | 0.112 |
| Astragaloside Ⅳ | 4.93 | 807.0 | 627.0 | + | 0.020 |
| Astragaloside I | 5.02 | 891.4 | 711.4 | + | 0.289 |
| Germacrone | 5.19 | 219.0 | 201.0 | + | 0.459 |

**Supplementary Table S2 The relative standard deviations (RSD%) of 47 bile acids.**

| **NO.** | **Analytes** | **Abbreviation** | **Representative** | R2 | **Linear Range** | **RSD%** |
| --- | --- | --- | --- | --- | --- | --- |
|  |  |  | **Regression Equation** |  | **(ng/mL)** |  |
| 1 | Glycocholic acid | GCA | y = 0.01301 x + 5.89915e-4 | 0.9952 | 0.20-1000.00 | 2.75 |
| 2 | Glycochenodeoxycholic acid | GCDCA | y = 0.01448 x + 2.35117e-4 | 0.9919 | 0.20-1000.00 | 3.08 |
| 3 | Taurocholic acid | TCA | y = 0.01320 x + 3.44161e-4 | 0.9936 | 0.10-1000.00 | 2.75 |
| 4 | Taurochenodeoxycholic acid | TCDCA | y = 0.01991 x + 0.00444 | 0.9923 | 0.50-1000.00 | 2.51 |
| 5 | Glycoursodeoxycholic acid | GUDCA | y = 0.03236 x + 0.00830 | 0.9939 | 0.50-1000.00 | 1.63 |
| 6 | Allocholic Acid | ACA | y = 0.01020 x + 0.00223 | 0.9919 | 1.00-500.00 | 3.95 |
| 7 | Ursodeoxycholic acid | UDCA | y = 0.00634 x + 9.79589e-4 | 0.9944 | 0.50-1000.00 | 4.8 |
| 8 | Deoxycholic acid | DCA | y = 0.00739 x + 0.00169 | 0.9925 | 0.20-1000.00 | 3.09 |
| 9 | Tauroursodeoxycholic acid | TUDCA | y = 0.01928 x + 7.41870e-4 | 0.9945 | 0.10-1000.00 | 7.16 |
| 10 | Hyodeoxycholic acid | HDCA | y = 0.00667 x + 0.00249 | 0.9924 | 0.50-1000.00 | 2.1 |
| 11 | apocholic acid | apoCA | y = 0.00458 x + 0.00276 | 0.9916 | 1.00-1000.00 | 4.38 |
| 12 | Glycodeoxycholic acid | GDCA | y = 0.05615 x + 0.00557 | 0.9922 | 0.20-1000.00 | 2.89 |
| 13 | Glycolithocholic acid | GLCA | y = 0.05615 x + 0.00557 | 0.9924 | 0.50-1000.00 | 3.17 |
| 14 | Alpha-Muricholic acid | α-MCA | y = 0.00116 x + 2.72698e-4 | 0.991 | 0.50-1000.00 | 8.97 |
| 15 | Beta-Muricholic acid | β-MCA | y = 7.15511e-4 x + 6.65815e-4 | 0.991 | 2.00-1000.00 | 6.44 |
| 16 | 7-ketoLithocholic acid | 7-KLCA | y = 0.01000 x + 2.47557e-4 | 0.9947 | 0.10-1000.00 | 4.94 |
| 17 | Tauro-α-muricholic acid | T-α-MCA | y = 0.02639 x - 2.08700e-4 | 0.9944 | 0.10-1000.00 | 4.73 |
| 18 | Tauro-β-muricholic acid | T-β-MCA | y = 0.03040 x - 2.59327e-4 | 0.9952 | 0.10-1000.00 | 4.66 |
| 19 | Omega-Murichoclic acid | ω-MCA | y = 0.00344 x + 0.00186 | 0.9921 | 1.00-1000.00 | 4.84 |
| 20 | murideoxycholic acid | MDCA | y = 0.01774 x + 5.10940e-4 | 0.9949 | 0.20-1000.00 | 6.52 |
| 21 | Taurohyodeoxycholic acid | THDCA | y = 0.03787 x + 0.00299 | 0.9913 | 0.20-1000.00 | 4.83 |
| 22 | Taurohyocholic acid | THCA | y = 0.05820 x + 0.00252 | 0.9965 | 0.10-1000.00 | 3.91 |
| 23 | taurolithocholic acid | TLCA | y = 0.02249 x + 0.00121 | 0.9906 | 0.20-1000.00 | 4.69 |
| 24 | Taurodeoxycholate acid | TDCA | y = 0.02039 x + 0.00112 | 0.996 | 0.10-1000.00 | 3.13 |
| 25 | Lithocholic acid | LCA | y = 0.00210 x + 0.00356 | 0.9915 | 5.00-1000.00 | 6.06 |
| 26 | Cholic acid | CA | y = 0.00689 x + 0.00203 | 0.9922 | 1.00-1000.00 | 4.27 |
| 27 | Chenodeoxycholic acid | CDCA | y = 0.01011 x + 0.00537 | 0.9909 | 0.50-1000.00 | 3.76 |
| 28 | hyocholic acid | HCA | y = 0.00342 x + 6.15178e-4 | 0.9931 | 0.50-1000.00 | 7.16 |
| 29 | norcholic acid | NorCA | y = 0.00562 x + 6.69526e-5 | 0.9927 | 0.20-1000.00 | 2.55 |
| 30 | Glycohyocholic acid | GHCA | y = 0.01287 x + 8.84143e-4 | 0.9927 | 0.10-1000.00 | 2.45 |
| 31 | 23-Nordeoxycholic acid | NorDCA | y = 0.00408 x + 7.18824e-5 | 0.9977 | 0.20-1000.00 | 3.11 |
| 32 | isolithocholic acid | isoLCA | y = 0.00387 x - 5.56792e-4 | 0.9929 | 1.00-1000.00 | 5.73 |
| 33 | 12-ketolithocholic acid | 12-KLCA | y = 0.00815 x + 0.00143 | 0.9912 | 0.50-1000.00 | 4.61 |
| 34 | dehydrolithocholic acid | DHLCA | y = 0.03538 x + 0.01694 | 0.9926 | 0.50-1000.00 | 6.41 |
| 35 | Lithocholic acid 3-sulfate | LCA-3S | y = 0.03458 x + 0.00399 | 0.9913 | 0.20-1000.00 | 1.99 |

| **NO.** | **Analytes** | **Abbreviation** | **Representative** | R2 | **Linear Range** | **RSD%** |
| --- | --- | --- | --- | --- | --- | --- |
|  |  |  | **Regression Equation** |  | **(ng/mL)** |  |
| 36 | Chenodeoxycholic acid-3-β-D-glucuronide | CDCA-3Gln | y = 0.02896 x + 4.97659e-4 | 0.9916 | 0.10-1000.00 | 4.84 |
| 37 | 3β-Ursodeoxycholic acid | 3β-UDCA | y = 0.00589 x + 0.00308 | 0.9917 | 0.50-1000.00 | 3.86 |
| 38 | 3-Dehydrocholic acid | 3-DHCA | y = 0.01374 x + 3.49861e-4 | 0.9949 | 0.20-1000.00 | 2.16 |
| 39 | Chenodeoxycholic Acid 24-Acyl-β-D-glucuronide | CDCA-24A-βGlu | y = 0.00787 x + 0.00382 | 0.9921 | 0.50-1000.00 | 6.29 |
| 40 | 12-ketochenodeoxycholicacid | 12-KCDCA | y = 0.00122 x + 4.22390e-4 | 0.9954 | 1.00-1000.00 | 7.01 |
| 41 | 7,12-Diketolithocholic acid | 7,12-DKLCA | y = 6.25547e-4 x + 2.98845e-4 | 0.9911 | 1.00-1000.00 | 9.02 |
| 42 | Dehydrocholic acid | DHCA | y = 0.00408 x + 3.74742e-4 | 0.9933 | 0.20-1000.00 | 5.3 |
| 43 | Ursocholic acid | UCA | y = 0.01162 x + 6.61963e-4 | 0.9919 | 0.20-1000.00 | 2.77 |
| 44 | 7-ketodeoxycholic acid | 7-DHCA | y = 0.00430 x + 5.34993e-5 | 0.9936 | 0.20-1000.00 | 2.51 |
| 45 | Isodeoxycholic acid | isoDCA | y = 0.00430 x + 5.34993e-5 | 0.9914 | 2.00-1000.00 | 6.93 |
| 46 | 3β-Cholic acid | βCA | y = 0.01273 x + 2.60833e-4 | 0.9945 | 0.10-1000.00 | 1.86 |
| 47 | Tauro-ω-muricholic acid | T-ω-MCA | y = 0.01273 x + 2.60833e-4 | 0.9956 | 0.10-1000.00 | 4.45 |

**Supplementary Table S3 49 top differentially abundant genera.**

| **NO.** | **Name** | **Sham-Mean(%)** | **Model-Mean(%)** | **AC-Mean(%)** | **P_value** |
| --- | --- | --- | --- | --- | --- |
| 1 | *norank_f__Muribaculaceae* | 20.91 | 6.116 | 4.306 | 0.0249 |
| 2 | *Citrobacter* | 0.00083 | 13.76 | 9.305 | 0.0029 |
| 3 | *norank_f__norank_o__Clostridia_UCG-014* | 15.23 | 1.715 | 2.85 | 0.0164 |
| 4 | *Enterococcus* | 0.01774 | 6.443 | 7.708 | 0.0045 |
| 5 | *Escherichia-Shigella* | 0.00055 | 4.766 | 5.482 | 0.0040 |
| 6 | *norank_f__norank_o__Clostridia_vadinBB60_group* | 0.2021 | 5.265 | 0.8537 | 0.0454 |
| 7 | *Staphylococcus* | 0.00811 | 0.4556 | 4.274 | 0.0033 |
| 8 | *norank_f__norank_o__RF39* | 2.927 | 0.01047 | 0.7385 | 0.0098 |
| 9 | *Enterorhabdus* | 1.482 | 0.1242 | 1.353 | 0.0117 |
| 10 | *Rikenellaceae_RC9_gut_group* | 1.761 | 0.2399 | 0.6746 | 0.0215 |
| 11 | *Ruminococcus* | 1.976 | 0.1029 | 0.01134 | 0.0074 |
| 12 | *Candidatus_Arthromitus* | 0 | 1.546 | 0.519 | 0.0081 |
| 13 | *Eubacterium_siraeum_group* | 1.794 | 0.00083 | 0.203 | 0.0072 |
| 14 | *Streptococcus* | 0.01832 | 0.09043 | 1.74 | 0.0047 |
| 15 | *Prevotellaceae_NK3B31_group* | 0.9589 | 0.5533 | 0.00842 | 0.0108 |
| 16 | *Eubacterium_xylanophilum_group* | 0.7191 | 0.0567 | 0.5914 | 0.0267 |
| 17 | *norank_f__Oscillospiraceae* | 0.6126 | 0.07298 | 0.4562 | 0.0331 |
| 18 | *Lachnoclostridium* | 0.5609 | 0.02501 | 0.3466 | 0.0185 |
| 19 | *Adlercreutzia* | 0.4062 | 0 | 0.1573 | 0.0042 |
| 20 | *Lachnospiraceae_UCG-006* | 0.2646 | 0.01948 | 0.07676 | 0.0218 |
| 21 | *Muribaculum* | 0.3222 | 0.01221 | 0.01948 | 0.0073 |
| 22 | *Lachnospiraceae_UCG-001* | 0.1506 | 0.00318 | 0.1614 | 0.0243 |
| 23 | *NK4A214_group* | 0.166 | 0.02239 | 0.08316 | 0.0221 |
| 24 | *Monoglobus* | 0.1887 | 0.01105 | 0.06484 | 0.0122 |
| 25 | *unclassified_f__Eggerthellaceae* | 0.03751 | 0 | 0.1527 | 0.0192 |
| 26 | *unclassified_f__Erysipelotrichaceae* | 0.02791 | 0 | 0.1204 | 0.0471 |
| 27 | *norank_f__norank_o__norank_c__Clostridia* | 0.1212 | 0.00756 | 0.01512 | 0.0068 |
| 28 | *Family_XIII_AD3011_group* | 0.1026 | 0.02646 | 0.00756 | 0.0118 |
| 29 | *Eubacterium_brachy_group* | 0.07531 | 0.0125 | 0.03402 | 0.0193 |
| 30 | *norank_f__Christensenellaceae* | 0.06978 | 0.0221 | 0.02733 | 0.0397 |
| 31 | *norank_f__Erysipelatoclostridiaceae* | 0.09159 | 0.01047 | 0.00955 | 0.0403 |
| 32 | *unclassified_f__Oscillospiraceae* | 0.06542 | 0.00781 | 0.02937 | 0.0266 |
| 33 | *Eubacterium_nodatum_group* | 0.06688 | 0.01599 | 0.01483 | 0.0171 |
| 35 | *UCG-005* | 0.06804 | 0.00431 | 0.01628 | 0.0081 |
| 35 | *UCG-003* | 0.07095 | 0.00144 | 0.00431 | 0.0043 |
| 36 | *Anaerofustis* | 0.04827 | 0.005815 | 0.01047 | 0.01508 |

| **NO.** | **Name** | **Sham-Mean(%)** | **Model-Mean(%)** | **AC-Mean(%)** | **P_value** |
| --- | --- | --- | --- | --- | --- |
| 37 | *Anaeroplasma* | 0.05263 | 0 | 0.004071 | 0.005679 |
| 38 | *Defluviitaleaceae_UCG-011* | 0.03547 | 0.0125 | 0.006688 | 0.02762 |
| 39 | *norank_f__Caulobacteraceae* | 0.001745 | 0.01105 | 0.02006 | 0.03236 |
| 40 | *UBA1819* | 0.02472 | 0.0002908 | 0.006688 | 0.007283 |
| 41 | *norank_f__Eggerthellaceae* | 0.02035 | 0 | 0.01018 | 0.01189 |
| 42 | *Parvibacter* | 0.01279 | 0.0002908 | 0.01134 | 0.009926 |
| 43 | *Ruminiclostridium* | 0.01338 | 0.007269 | 0.0005815 | 0.02449 |
| 44 | *Turicibacter* | 0 | 0.004943 | 0.01396 | 0.01856 |
| 45 | *Tyzzerella* | 0.006397 | 0 | 0.005815 | 0.02355 |
| 46 | *norank_f__Coriobacteriales_Incertae_Sedis* | 0.007269 | 0.001163 | 0.002326 | 0.02085 |
| 47 | *norank_f__norank_o__Oscillospirales* | 0.005815 | 0.0005815 | 0.001454 | 0.007895 |
| 48 | *Solibacillus* | 0 | 0 | 0.004361 | 0.0345 |
| 49 | *Sphingomonas* | 0.0002908 | 0.0002908 | 0.002908 | 0.01943 |

**Supplementary Table S4 15 genes involved in the secondary bile acid biosynthesis pathway**

| **NO.** | **KO** | **Genes** | **Sham-Mean(%)** | **Model-Mean(%)** | **AC-Mean(%)** | **P-value** |
| --- | --- | --- | --- | --- | --- | --- |
| 1 | K22604 | FabG | 0.1246 | 0.09331 | 0.08209 | 0.02601 |
| 2 | K22605 | baiA | 0.02256 | 0.002673 | 0.01267 | 0.01611 |
| 3 | K15868 | baiB | 0.1142 | 0.09421 | 0.07308 | 0.01195 |
| 4 | K00076 | hdhA | 0.7136 | 0.519 | 0.6783 | 0.3855 |
| 5 | K23231 | fabG_2 | 0.55 | 0.4232 | 0.4707 | 0.2636 |
| 6 | K22606 | NAD+ | 0.3621 | 0.2398 | 0.2766 | 0.5805 |
| 7 | K22607 | NADP+ | 0.3627 | 0.2367 | 0.2733 | 0.1845 |
| 8 | K01442 | cbh | 0.6031 | 0.6667 | 0.6508 | 0.5671 |
| 9 | K15871 | baiF | 0.3226 | 0.267 | 0.2442 | 0.1394 |
| 10 | K15869 | fabG_2 | 0.3099 | 0.2057 | 0.2452 | 0.119 |
| 11 | K15870 | baiCD | 0.2976 | 0.1841 | 0.2657 | 0.2963 |
| 12 | K15872 | baiE | 0.3034 | 0.1718 | 0.2239 | 0.0667 |
| 13 | K15873 | baiH | 0.1753 | 0.2453 | 0.2486 | 0.2199 |
| 14 | K15874 | baiI | 0.2645 | 0.172 | 0.2297 | 0.2086 |
| 15 | K07007 | baiN | 0.214 | 0.1663 | 0.2185 | 0.3679 |
